# Diagnosis and Management of Malignant Epithelial Nail Unit Tumors

**DOI:** 10.3390/diagnostics14212379

**Published:** 2024-10-25

**Authors:** Matilde Iorizzo, Laura Vollono, Bertrand Richert

**Affiliations:** 1 Private Dermatology Practice, 6500 Bellinzona, Switzerland; 2 Private Dermatology Practice, 00196 Rome, Italy; laura.vollono@gmail.com; 3 Dermatology Department, Saint Pierre and Brugmann University Hospitals, Université Libre de Bruxelles, 1000 Brussels, Belgium; bertrand.richert@chu-brugmann.be

**Keywords:** nail tumor, malignant nail tumor, management, treatment, surgery, squamous cell carcinoma, Bowen disease, malignant onychopapilloma, onychocytic carcinoma, onycholemmal carcinoma

## Abstract

**Background**: Malignant epithelial nail unit tumors pose significant diagnostic and therapeutic challenges due to their clinical presentation often mimicking benign conditions and due to the need to preserve as much nail unit function as possible during surgery. Early detection is crucial, even if none of these tumors represent a life-threatening disease. **Objectives**: This review focus on squamous cell carcinoma, verrucous carcinoma, eccrine porocarcinoma, onychocytic carcinoma, basal cell carcinoma, malignant onychopapilloma, malignant onycholemmal cyst and onycholemmal carcinoma. **Methods**: Existing literature on the aforementioned tumors has been revised and synthesized. **Results**: Clinical presentation, pathology, diagnostic procedures, risk factors and the challenges associated with surgical management have been described in detail. **Conclusions**: Malignant epithelial tumors of the nail unit require careful evaluation and management due to their complex presentation. Early detection and an informed surgical approach are essential to improve patient outcomes and minimize complications.

## 1. Introduction

Malignant epithelial nail unit tumors, though relatively rare (besides squamous cell carcinoma), present a diagnostic and therapeutic challenge due to their clinical presentation and the critical nail unit functions that should be surgically preserved to the greatest extent possible without affecting the prognosis. Nail tumors, in fact, usually present clinical signs that mimic benign conditions, leading to delayed diagnosis. This is due to the fact that the nail apparatus has a limited repertoire of reaction patterns. A tumor should be always suspected when dealing with a unexplained single-digit dystrophy that is unresponsive to treatment, especially if it is characterized by ulceration and/or bleeding. Pain or discomfort as well as periungual soft tissue abnormalities and nail plate color changes should also be taken into account. It is important not to forget that nail unit melanoma is not the only pigmented tumor; some of the epithelial tumors may also present with a brown pigmentation. All these signs may be present individually, be associated with time, or be present together at the time of diagnosis—this depends on the type of tumor and its evolution over time.

Understanding the suspicious clinical features, risk factors, and management strategies of malignant epithelial nail unit tumors is essential for improving patient outcomes and minimizing morbidity. This paper aims to summarize the main features of the following tumors: squamous cell carcinoma, verrucous carcinoma, eccrine porocarcinoma, onycocitic carcinoma, basal cell carcinoma, malignant onychopapilloma, malignant onycholemmal cyst and onycholemmal carcinoma.

## 2. Diagnosis

### 2.1. Squamous Cell Carcinoma

Squamous cell carcinoma (SCC) is the most common malignant nail tumor [1], where the in situ form (Bowen disease) is more common than the invasive one. Risk factors include trauma, ionizing radiation, oral exposure to arsenic or pesticides and dyskeratosis congenita. Human papillomavirus (HPV) infection has been demonstrated to play a major role in SCC pathogenesis, being detected in up to 60% of cases. Serotype 16 is the most frequently identified (75%), followed by serotypes 2, 6, 11, 18, 26, 31, 34, 35, 56, 58 and 73 [2,3,4,5,6,7,8,9]. HPV-positive cells have been found even in perilesional skin, suggesting that HPV infection spreads beyond the clinically visible lesion [10]. This may explain the high recurrence rate of this tumor. Almost one-third of patients with SCC of the nail apparatus have a history of HPV-associated genital disease, such as genital warts, dysplasia or cancer of the cervix or anogenital region, or a sexual partner with such a history [3,10]. The same HPV type was found to be concordant in 10% of patients with genital and digital diseases, with the genital condition preceding nail unit disease by approximately 12 years [3,11]. For these reasons, it has been proposed that SCC of the nail unit is a hidden high-risk HPV-associated reservoir and should be recognized as a sexually transmitted infection [1,10,11,12].

The condition generally occurs during late adulthood, with a peak incidence between the ages of 50 and 69 years and a higher prevalence among men compared to women (2:1 ratio) [2]. Darker phototypes (IV–VI) seem to have an earlier diagnosis, in terms of age, compared to lighter skin types. Fingernails are most commonly affected, especially the index and middle finger of the right (dominant) hand [13,14,15,16]. SCC is mainly a monodactylic condition, but polydactylic forms have also been reported, especially in immunosuppressed patients [2,17,18,19].

The malignant process, which is usually indolent and painless, may develop in the epithelium of the periungual area (31.5%), in the nail bed (57.4%), or both (11%) [2,10]. It is not unusual for lesions to have been present for years, sometimes for more than a decade, before being diagnosed [20]. Patients often do not care and seek medical advice very late (after 6 years on average) [2,3,21]. Moreover, SCC is very often misdiagnosed by non-expert physicians, resulting in misguided treatments and a delay in the correct diagnosis. Data from a Belgian third-level referral center reported that SCC was suspected only in 12.5% in patients referred from other physicians, while dermatologists with expertise in nail diseases correctly hypothesized a diagnosis of SCC in 76.6% of cases [2].

#### 2.1.1. Clinical Features

SCC displays a wide range of clinical patterns according to its location in the nail apparatus and its level of invasiveness. For this reason, it is also called “the great mimicker”. Lesions are clinically classified into two main categories: periungual type (PUT), arising in the epithelium of the nailfold and nail groove, and subungual type (SUT), developing in the nail bed epithelium.

**Figure 1:**
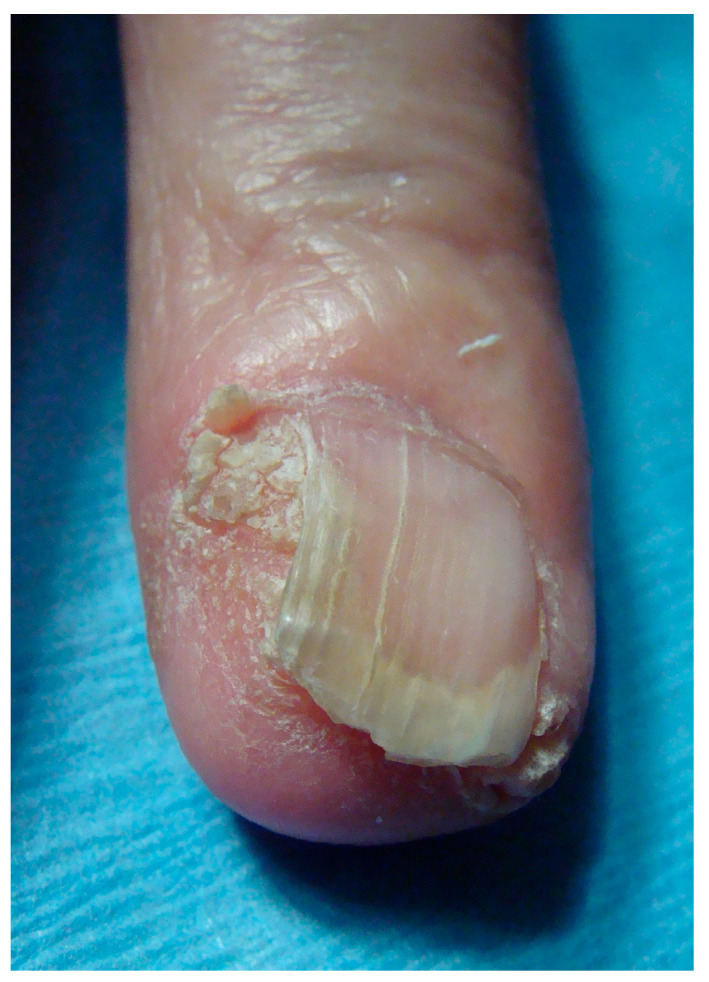
Bowen disease arising in the periungual area and presenting as a warty hyperkeratosis associated with nail plate dystrophy and onycholysis. This presentation, which is very common, is often misdiagnosed as a viral wart.

**Figure 2:**
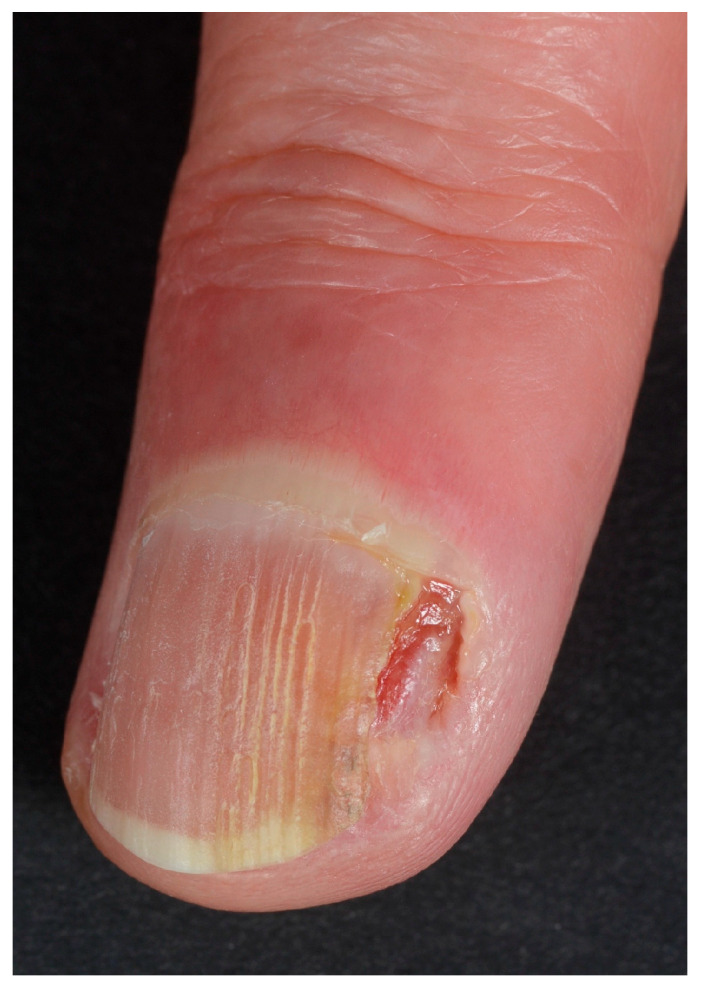
SCC arising in the periungual area and presenting with erosion and oozing. Both these signs are suspicious, especially if the patient is not able to refer any prior trauma (image courtesy of M.C. Pasch, Nijmegen, NL).

When arising in the periungual area (PUT), the tumor presents mainly as a warty hyperkeratosis associated with scaling, possible nail plate pigmentation, and sometimes erosion, but rarely ulceration (Figure 1 and Figure 2). Very commonly, it extends deeply in the lateral groove and sometimes to the very lateral part of the nail bed, explaining why topical treatments do not work in this location—they are unable to reach the deep portion of the tumor. A treacherous clinical sign is periungual swelling, from either deep tumor proliferation or inflammatory paronychia, and this should always lead to suspicion that one may be dealing with an SCC. The main differential diagnosis is of a viral wart, especially when erosion and oozing are not present. The clinical presentation of in situ or invasive SCC is similar in this area, and only histopathological examination will allow the differential diagnosis. PUT is strongly associated with HPV infection, especially that of HPV16 and HPV73 [22,23].

**Figure 3:**
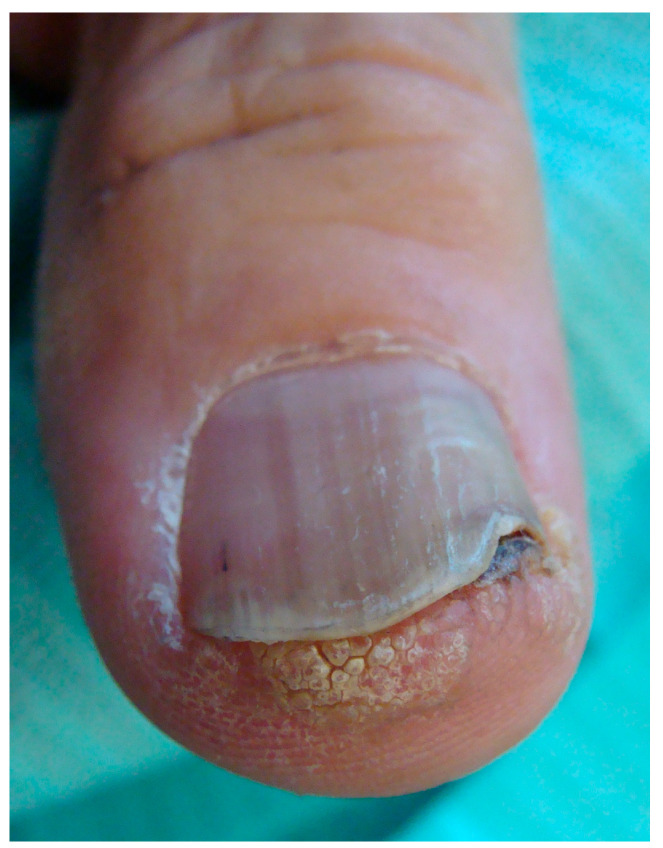
Hyperkeratotic SSC developed in the nail bed epithelium and extending to the hyponychium—this clinical presentation strongly mimics a wart.

**Figure 4:**
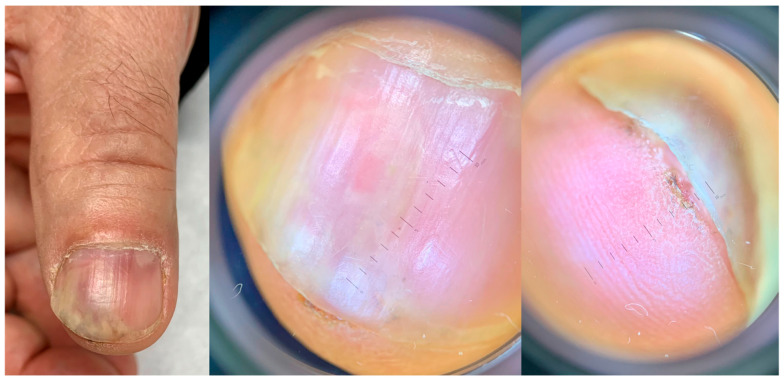
SCC presenting with onycholysis and nail bed oozing. The nail bed redness spotted through the nail plate is highly suggestive of abnormal vascularity and makes it mandatory to remove the plate to better observe the area.

**Figure 5:**
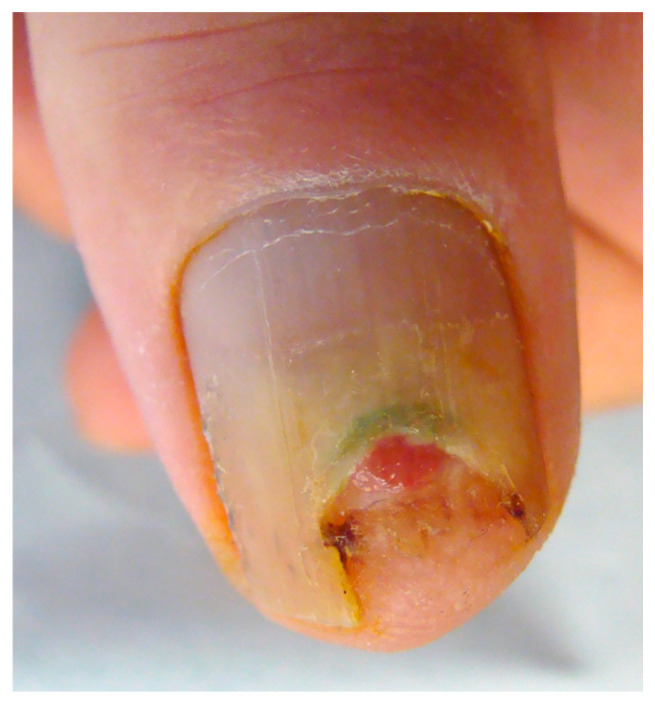
SCC presenting as a vascular mass in the nail bed mimicking a pyogenic granuloma.

**Figure 6:**
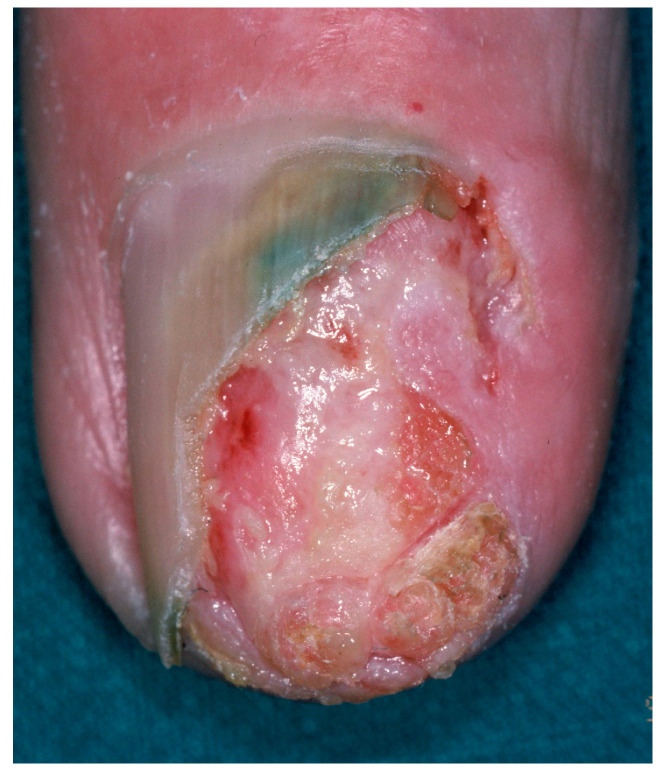
SCC of the nail bed with extensive erosions and ulcerations—the onycholytic plate has been clipped away to better show what is underneath. An amelanotic melanoma should be ruled out with such a clinical presentation.

**Figure 7:**
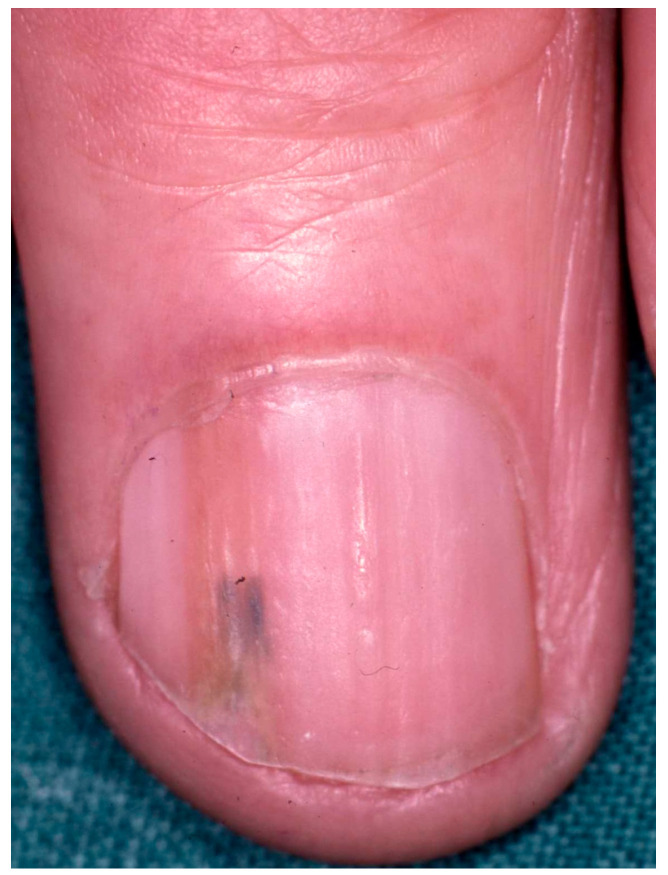
Bowen disease presenting as longitudinal erythronychia. Onychopapilloma is probably the disease most commonly differentially diagnosed as a result of such a presentation.

**Figure 8:**
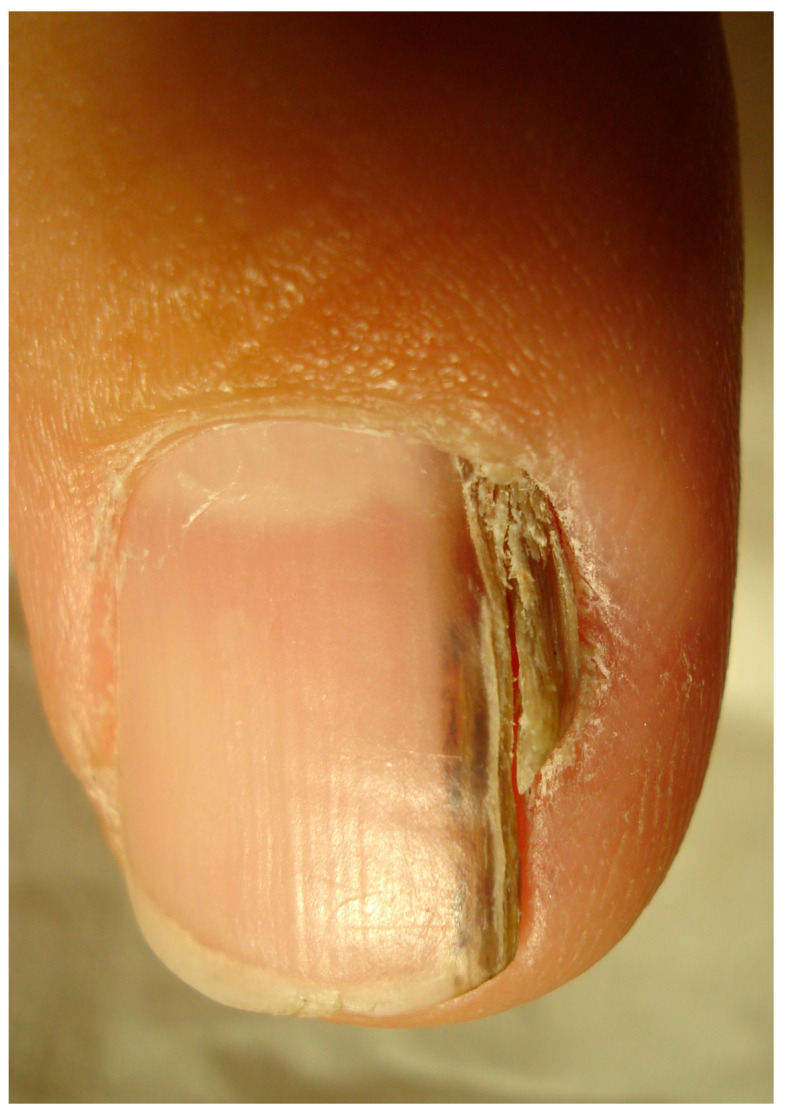
Bowen disease presenting as longitudinal melanonychia. This clinical presentation is highly suspicious for SCC and deserves further investigation.

When arising in the subungual area (SUT), the tumor presents different clinical signs that are always associated with onycholysis (Figure 3, Figure 4, Figure 5 and Figure 6) [24,25,26]. It may also show nail plate dystrophy, localized or diffused hyperkeratosis of the nail bed, polymorphous vascular structures, erosion, ulceration and oozing. A simple red (erythronychia) (Figure 7) or brown (melanonychia) (Figure 8) longitudinal band is also a possible presentation. The presence of a longitudinal melanonychia along with a hyperkeratosis of the lateral fold and possible plate dystrophy represents a red flag and should immediately arouse suspicion of Bowen’s disease [27,28], but other pigmented tumors should be considered (onychomatricoma or acanthoma) in differential diagnosis as well as pigmented onychomycosis. Oozing is a result of ulceration of the nail bed and also represents a red flag for a suspicious subungual lesion. Its presence should always be investigated further by clipping away the portion of detached nail plate. This clinical sign, however, may also result from a bacterial infection or a pyogenic granuloma. Oozing is an important but underrecognized sign, which is only occasionally reported in the literature. It may be the only sign of a subungual malignancy and should never be overlooked. In SUT, the prevalence of HPV and its different serotypes is still an evolving subject. While an association with HPV16 has been reported in the past, a recent study reported an absence of infection in 100% of cases [10,23]. A subset of SUTs in situ clinically presenting with longitudinal melanonychia has been associated with HPV56 and with HPV73 infection due to their particular affinity for the subungual epithelium [4,29].

#### 2.1.2. Dermoscopic Features

The dermoscopic appearance of PUT is similar to that of in situ SCC located elsewhere on the skin but also shares some characteristics with viral warts. Scaling and roughness have been reported in all studies, while ulceration was rarely observed. Up to 5.5% cases of in situ tumors contain pigment. In such cases, structureless gray to brown pigmentation, linear arrangement of small brown or gray dots, and peripheral out-of-focus radial streaks have been observed [30,31,32]. In addition to pigmentation, dermoscopy also helps to detect minimal nail plate dystrophies (with disrupted margins above all). Typical vascular structures may be noticed on the lateral fold, as in superficial SCC on the skin elsewhere, even if most of the time the hyperkeratosis does not allow us to see them.

**Figure 9:**
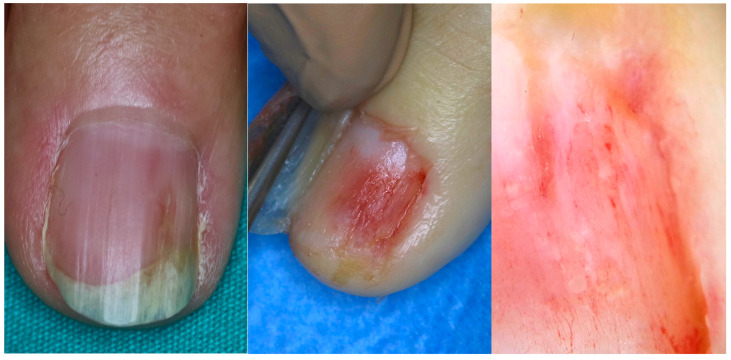
SCC presenting with nail bed dotted vessels and vessels clustered in groups (image courtesy of F. Goktay, Istanbul, TR).

In SUT, when there is no warty presentation, vascular structures, better observed with magnifications from 50 to 70× and after nail plate removal, are the most relevant: dot, linear, hairpin and glomerular vessels have been reported [33,34]. The vascular pattern of invasive forms is of special interest and it is characterized by dot-like to glomerular, looped vessels clustering in groups (Figure 9) [35].

Nail plate removal is, however, not always feasible and dermoscopic examination showing splinter hemorrhages, whitish or yellowish longitudinal lines, distal onycholysis with polycyclic/fuzzy edges and short-segmented longitudinal erythronychia is not helpful for diagnosis because these signs are not specific—they can be present as a result of many other conditions, including inflammatory and traumatic nail disorders. Examination of the free distal margin may instead reveal roughness underneath the nail plate and, above all, oozing [36]—these are suspicious signs that deserve further attention.

Intraoperative dermoscopy has been shown to be helpful to better define tumor margins [37]. Nail plate removal before surgery has revealed, according to different reports, white-gray reticular patches over a bright red nail bed (vascular dots), non-parallel side edges and yellowish scales [38]. Analysis of the vascular pattern is the key because, as stated above, it exhibits polymorphous features [36,37,38]. A polymorphic vascular pattern was found to be significantly correlated with a deeper invasion of SCC [36].

#### 2.1.3. Histopathological Features

Histology of SCC of the nail unit shows typical characteristics of cutaneous SCC: loss of normal stratification throughout all epidermal layers, dyskeratosis, large clumped cells with hyperchromatic nuclei and atypical mitoses with possible perinuclear vacuolization, which is indicative of association with HPV [20,39]. The differential diagnosis between in situ and invasive forms is the intact basement membrane defining the in situ form. As with many other potentially invasive tumors, it is possible that a biopsy performed for diagnostic purposes may reveal only an in situ form when the whole tumor presents invasive clusters. This is something that is always important to remember before surgical planning.

Recently, two main histopathological types of SCC have been identified based on epithelium color at low-power microscopic examination and correlated with different clinical presentations [23]:

(a) A blue basaloid pattern—characterized by basaloid cells, it presents with two different subtypes: one with flat epithelium, composed of keratinocytes with scant cytoplasm with hyperchromatic, basophilic nuclei and full-thickness mitoses, and a verrucous one composed of keratinocytes with enlarged eosinophilic cytoplasms, atypical hyperparakeratosis, prominent atypia and koilocytosis. The basaloid pattern has been associated with abnormal p53 expression and the presence of HPV infection. It is mainly exhibited by in situ SCC and usually appears in the periungual area of a younger population.

(b) A pink keratinizing pattern—characterized by a flat or ulcerated epithelium composed by keratinocytes with vesicular nuclei and eosinophilic cytoplasm and marked dyskeratosis, which leads to its hypereosinophilic pink appearance. It presents elongation of the rete ridges, loss of cellular cohesion and cytoid bodies, with no koilocytosis. The keratinizing pattern has been significantly associated with overexpression of the proliferation marker Ki67 and no association with HPV infection has been found. It is mainly exhibited by invasive SCC and usually presents in the subungual area of elderly people.

More studies regarding the correlation between histopathological presentation, clinical presentation and HPV infection could provide valuable information for the correct management of these patients. Next-generation sequencing (NGS) has been used to obtain a molecular characterization of SCC samples with the aim of identifying the possible genetic drivers involved in this tumor. TP53 was the most mutated gene (6/20 cases, 30.0%), while cKit, GNAS, EGFR, DICER1 and CTNNB1 were observed in one sample each (5.0%). No parameters were associated with the mutational status [40].

#### 2.1.4. Image Analysis

Radiological examination is usually performed to detect locoregional metastases including bone invasion. A recent study showed that imaging help guiding patient management but only histology has been proven to be right as sometimes nothing is visible on X-rays [41]. In SCC, bony involvement is observed in less than 20% of cases [24] and its presence is important to detect because it compels amputation [42] or, as more recently proposed, tangential avulsion of the distal phalanx [43].

Magnetic image resonance (MRI) is more useful because it allows us to differentiate between edema due to inflammation or tumor compression against the periosteum and bone invasion. It should be performed before surgery, otherwise it will not be possible to differentiate between post-surgery inflammatory changes and primary edema/bone involvement. In a study on a small number of cases, interestingly, MRI showed that the deep margin of the tumor with the dermis was always well defined for Bowen’s disease and blurred for invasive SCC [44]. MRI can also support early detection of subclinical relapses. It is to be noted that most commonly available MRI devices vary between 1.5 Tesla and 3.0 Tesla. The higher the number of Tesla units, the higher the axial spatial resolution. For this reason, MRIs operating at 3.0 Tesla, although not specifically dedicated to small areas, are often those advisable for nails [45].

High-frequency ultrasounds (HFUSs) might be a less expensive first step before evaluating the need for MRI. HFUSs can provide insights into location, size, shape, echogenicity, vascularization (hypervascularity), infiltrative nature (the presence of necrotic areas, bone cortical invasion) and subclinical relapses without the need to inject a contrast media [45]. To date, HFUS is not able to detect pigment. Linear high-frequency probes (15–33 MHz) are usually those preferred in the initial evaluation, since they are available worldwide; ultra-high-frequency probes (50–70 MHz) are better for superficial lesions or to obtain a better resolution but are available only in selected centers. At HFUS, SCC appears as a heterogeneously hypoechoic and hypervascular focal mass with a solid component and blurred and irregular margins, especially in its invasive forms. Bone invasion and focal necrosis are also possible to observe with this technique. In color Doppler mode, low-resistance pulsatile flow signals within the tumor or at the periphery are evident, usually with a chaotic pattern. These signs are, however, not specific for SCC, but are highly suggestive of a malignant tumor [41,46,47].

Reflectance confocal microscopy (RCM) can be helpful in discriminating between non-melanocytic vs. melanocytic lesions, as reported in one case of well-differentiated SCC presenting with a pigmented longitudinal band in the big toenail [48] (see Table 1). RCM provides detailed, high-resolution images allowing for the real-time visualization of cellular and structural changes within the nail unit. However, the difficulty in observing hyperkeratotic alterations, low penetration, low worldwide availability/high costs of the devices limit its use in this condition.

#### 2.1.5. Treatment and Prognosis

SCC can remain in situ for decades before its eventual progression to invasive SCC, which happens in 3–5% of cases [49]. SCC has been defined as a weakly aggressive tumor, with metastases reported in less than 10 cases worldwide [2].

The treatment of choice is micrographic Mohs surgery with three-dimensional assessment of the surgical margins [50,51]. The importance of this technique lies in the reduction in the number of unnecessary amputations, which is a critical consideration for patients’ quality of life [52]. Bony involvement, in fact, usually compels amputation of the distal phalanx even if a more conservative approach (tangential avulsion of the distal phalanx) has been recently proposed for superficial bone involvement [43]. Excision with surgical margins of 3–5 mm may be considered when Mohs surgery is not feasible or when the lesion involves less than 50% of the nail or it is restricted to its lateral portion. Recurrence after Mohs surgery is, however, significantly lower compared to traditional surgery, but varies according to different experiences (3.5% to 22%) [25,51,53,54,55]. Recurrence after traditional surgery has been correlated with periosteal invasion, which is usually associated with referred pain [53], but also with the viral origin of the condition [24].

Experiences with non-surgical treatments have also been reported in the literature. Photodynamic therapy with 5-aminolevulinic acid has demonstrated efficacy both in periungual tissue and on the nail bed after nail plate avulsion, but in very few cases [56,57,58,59]. Topical imiquimod and 5-fluoruracil have been used with good results in genital or extragenital in situ SCC; therefore, their use in the periungual area may appear as a good option [60,61]. Intra-arterial infusion with methotrexate has also been proposed [62]. However, the complex anatomy of the lateral fold allowing the HPV to hide deeply mainly explains why the tumor in the nail unit is usually not reachable in toto by these non-surgical approaches. None of them, in fact, allow for the controls of the tumor margins. These treatments should be considered as options for special cases when surgery is not feasible. Long-term follow-up is always mandatory and includes examination of all nails, investigation of pain and the biopsy of any suspicious changes. On average, these investigations should be performed every 3 to 6 months.

Due to a possible pathogenetic role of HPV, vaccines have become of special interest as a possible treatment option for SCC of the nail unit. The current approved vaccines are quadrivalent (HPVs 6, 11, 16 and 18), bivalent (HPVs 16 and 18) and 9-valent (HPVs 6, 11,16, 18, 31, 33, 45, 52 and 58) and are indicated for the prevention of invasive cervical cancer [63]. Although data are limited, therapeutic treatment has been investigated for cutaneous SCC with positive outcomes, resulting in a reduction in the number of dermatologic interventions following vaccination with 9-valent vaccine in immunosuppressed patients and complete clearance from cutaneous SCC lesions in an elderly patient who was not amenable to surgery [64,65]. Vaccination was either administered via intramuscular injection, intra-lesion, or in a combination of both. Only two case reports regarding the use of HPV vaccination in nail unit SCC are available to date. One reported complete resolution of polydactylous in situ SCC in a 15-year old female who underwent cryosurgery together with 9-valent vaccination, while another reported a 74-year-old man with a recurrent fingernail in situ SCC that received both intramuscular and intralesional injection of 9-valent vaccine with favorable results [66,67]. Such reports prompt more research. However, some limitations must be taken into consideration. While HPV16 infection accounts for the majority of HPV-related SCCs, vaccines do not cover all the HPV types associated with nail unit SCC.

### 2.2. Verrucous Carcinoma

Verrucous carcinoma is a highly keratinizing variant of SCC characterized by local aggressiveness but a low potential for metastasis [68]. The sole or the heel of the foot is the typical site of development of this tumor, where it is also known as carcinoma cuniculatum. It is exceptionally rare for verrucous carcinoma to originate in the nail bed. Along with SCC, this condition is difficult to promptly diagnose in a clinical setting and for this reason diagnosis is very often delayed, increasing the risk of invasion of the distal phalanx and distal interphalangeal joint [24].

#### 2.2.1. Clinical Features

**Figure 10:**
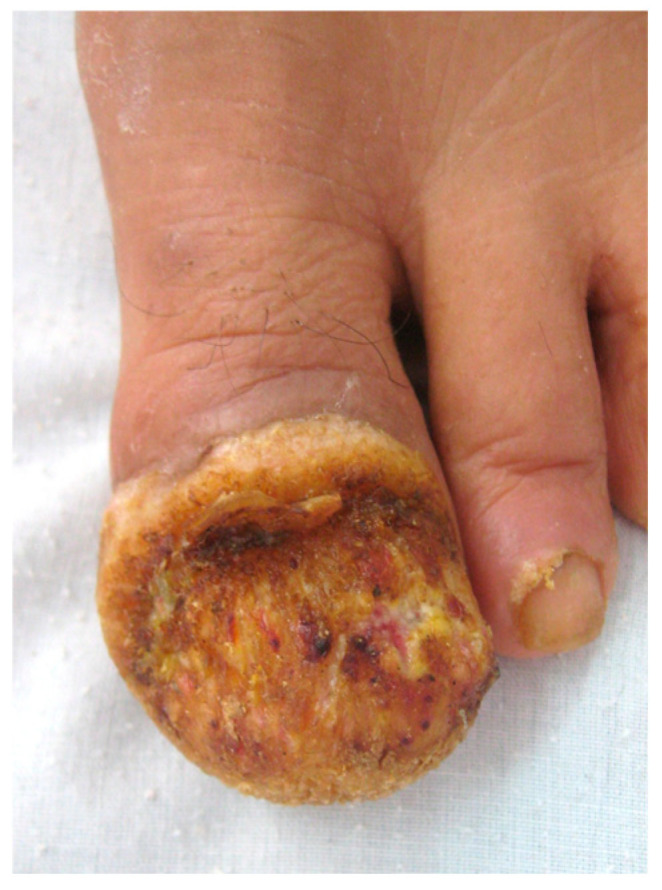
Verrucous carcinoma of the nail bed (image courtesy of S. Chiheb, Casablanca, MA).

The condition presents as a warty lesion with papillomatous digitations arising from the nail bed (Figure 10), most often involving the thumb, but also sometimes the great toenail and the fifth toenail in patients in their fifth or sixth decade of life [69,70,71]. The clinical differential diagnosis includes verruca vulgaris, SCC and porocarcinoma due to its warty aspect, but also amelanotic melanoma or a deep fungal or bacterial infection due to its destructive potential towards local tissues. No dermoscopy description of this tumor exists in its nail unit localization.

#### 2.2.2. Histopathological Features

Histopathological examination shows hyperkeratosis, parakeratosis and marked acanthosis, with the proliferating epithelium displaying a pushing border that compresses the adjacent connective tissue. Histopathological differentiation from wart or pseudoepitheliomatous hyperplasia can be challenging, due to fact that biopsies may often be too shallow to collect a specimen exhibiting substantial cytological atypia or infiltrative borders. Positivity for proliferation marker Ki67 and/or overexpression of p53 at immunohistochemistry have been described in verrucous carcinoma, often in basal cells and at the periphery of the neoplasm [69,70,71]. HPV is rarely isolated in verrucous carcinoma, but its presence has been detected in some cases [70].

#### 2.2.3. Imaging

As this tumor may affect the bone, radiological examination is usually performed to investigate bone invasion. No imaging method has been described in the literature, other than radiography always showing various grades of lytic bone lesions.

#### 2.2.4. Treatment and Prognosis

Verrucous carcinoma is locally aggressive, with constant destruction of the nail unit. Bone involvement has been reported in 36% of cases, prompting amputation when histologically confirmed. En bloc excision with appropriate margins followed by full-thickness graft or Mohs surgery are the treatment of choice when the bone is not involved [72].

Alternative treatments with imiquimod and with intra-arterial infusion of methotrexate have been reported, resulting in remission for more than 4 years [73]. External radiation has also been proposed as a salvage therapy for unresectable cases. Non-surgical treatments have, however, been associated with increased metastasis [72].

### 2.3. Eccrine Porocarcinoma

Eccrine porocarcinoma (EPC) is a rare malignant skin tumor arising from the intraepidermal ductal portion of the sweat gland [74]. Nail unit involvement is extremely unusual, with only eight cases published to date [75,76,77,78,79,80,81,82,83], and almost none reported a clinical image of such a tumor. It seems that EPC originates from the nail folds and then extends to the nail bed [84]. EPC was mostly reported on the first toenail (50%) or fourth finger (25%) of males (87.5%) over 70 years of age [78]. Multiple EPCs have been observed in patients with malignancies and in organ transplant recipients. This led to the proposal of a pathogenetic role of Merkel cell polyomavirus (MCPyV) as in Merkel cell carcinoma, but current opinion is that this virus is simply a passenger virus rather than an oncogenic driver. UV radiation does not seem to be a key pathogenetic point due to its origin from the deep dermis [85]. EPC develops within a pre-existing poroma in 18–50% of cases; hence, it has been proposed that EPC in situ could be the result of a transition from a poroma, due to the presence of residual poromas in a variable percentage of EPC in situ [74,86]. Both lesions exhibit similar mutation patterns, harboring activating mutations in HRAS or fusions of YAP/TAZ, YAP1-MAML2, YAP1-NUTM1 or WWTR1_NUTM1 [87].

#### 2.3.1. Clinical Features

The condition presents as an erythematous papule, nodule or plaque under the nail plate or arising on the lateral nailfold. The mass can be verrucous, eroded or ulcerated with onycholysis and possible dystrophy of the nail plate [78]. Pain is frequently reported [88]. Two cases of EPC presenting with erythema, swelling and granulation tissue of the lateral fold mimicking ingrown nail with pseudo-pyogenic granuloma have been reported [75,89]. Other diseases that should be evaluated during differential diagnosis are, subungual wart, SCC, exostosis, pyogenic granuloma and amelanotic melanoma.

#### 2.3.2. Dermoscopic Features

Only one report regarding dermoscopic features of EPC of the nail unit has been published and reports clusters of polymorphic vessels (globular, linear irregular and hairpin) on a pinkish background intersected with whitish structureless areas [76]. These findings are consistent with those of EPC arising in other anatomic locations where round-to-oval pinkish-white structureless areas and white-to-pink halos histologically correspond to a vascular and edematous stroma under the thin epidermis and mesh-like proliferation of tumor cells, respectively [90]. Such features have also been detected in poromas, but while in this condition they comprise the entirety of the lesion (“frog-egg pattern”), they are only focally observed in EPC [91,92]. A transitional dermatoscopic pattern characterized by whitish-pink areas in a diffuse distribution surrounded by pinkish-while halos (typical of EP) associated with central ulceration and polymorphous vascular pattern (EPC) has been observed in a case of in situ EPC originating from a previous poroma [92,93].

#### 2.3.3. Histopathological Features

EPC displays characteristics poromatous basaloid epithelial cells with ductal differentiation and significant cytological atypia. EPCs undergo a proliferative phase within the epidermis and may proceed to local invasion of the papillary and reticular dermis with potential for lymphovascular invasion and distant metastases [94].

#### 2.3.4. Imaging

No specific imaging is required for EPC, but it is advisable to perform an X-ray of the affected digit before any explorative surgery to exclude possible exostosis when the clinical presentation is reminiscent of this diagnosis.

#### 2.3.5. Treatment and Prognosis

EPC is a malignant lesion with the potential for local recurrence, lymph node invasion, and metastasis. The most frequent areas for distant metastasis are the lungs, liver, brain and skin and its prevalence ranges from 1% to 16.2% [95,96]. The prognosis of this tumor is, thus, very poor. Duration of the condition before diagnosis, elderly patients, tumor depth > 7 mm and lymphovascular invasion have been all associated with a negative outcome. Histologically, extensive pleomorphism, mitotic index, lymphovascular and perineural invasion, necrosis and epidermotropism are considered negative prognostic factors [74]. Tumor stage is, however, the most important prognostic factor [97].

Wide local excision is the treatment of choice for EPC arising on the skin, with margins ranging from <1 cm to >2 mm. At the nail unit, the recurrence rate is higher that of than EPC arising elsewhere on the skin (25% versus 11–20%) [78,98,99]. It might be complicated to obtain clear excision margins on partial removal of the nail unit [78] and this is the reason why amputation is more frequently performed (62.5%). Mohs micrographic surgery is probably an excellent solution at the nail unit when available. Local radiotherapy and chemotherapy are performed in metastatic disease [100,101].

### 2.4. Onychocytic Carcinoma

Onychocytic carcinoma is a rare (with less than 10 cases described) and well-demarcated epithelial tumor originating from the nail matrix epithelium. It exhibits a distinct picture of onychocytic differentiation, with signs of both nail matrix differentiation and nail plate differentiation. It is considered to be the malignant counterpart of onychocytic matricoma and has a low-grade malignancy.

#### 2.4.1. Clinical Features

The cases described present with a monodactylous acquired longitudinal thickening of the nail plate (xantho-leuco-pachyonychia) with an irregular surface. Transverse hypercurvature of the nail plate is observed, with the possible presence of cavities within the nail plate similar to those observed in onychomatricoma, but significantly smaller (<0.1 mm). The periungual tissues are spared. Clinical differential diagnoses primarily comprise other nail matrix tumors, especially onychomatricoma and onychocytic matricoma, which shares similar clinical patterns. However, no pigmented variants have been described for onychocytic carcinoma [20]. Dermoscopy features are also lacking.

#### 2.4.2. Histopathological Features

Histopathologically, onychocytic carcinoma is the prototype of a malignant onychomatrical neoplasm, showing a well-demarcated epithelial tumor with small finger-like epithelial protrusion with a regular prekeratogenous zone. These findings correspond to the small cavities observed clinically within the nail plate. In the deeper portion of the lesion basaloid cells with atypia and dyskeratosis are observed, usually with a low mitotic rate [102]. The expression of hair keratins, especially hair keratin 85, has been observed and may help histopathological differential diagnosis with SCC in situ [103]. Nail clipping microscopy may be useful for highlighting the typical honeycomb pattern with multiple small cavities described above and may allow other subungual malignant tumors to be ruled out [104,105].

#### 2.4.3. Treatment and Prognosis

No long-term surgical experience is available for this tumor due to its rarity and quite recent description. It appears to be a low-grade malignancy; thus, complete excision with histologically cleared margins is considered the treatment of choice. Larger series are needed in order to assess long-term prognosis.

### 2.5. Basal Cell Carcinoma

Basal cell carcinoma (BCC) is the most common human malignant neoplasm, accounting for approximately 75% of skin cancers. Conversely, it is the least common malignancy of the nail unit, with around 30 cases reported to date. It usually arises in the periungual area of patients over 60 years, mainly on the thumb or the big toe [24,106]. While the role of UV radiation has been established in the pathogenesis of BCC on the skin, the risk factors for nail unit BCC still need to be fully elucidated. Cases of nail unit BCC arising in the context of a periungual radiodermatitis, a habit tic dermatitis and that occurring as a consequence of accidental contact with an azo pigment have been described [107,108,109]. Nail polish was also suspected to be the cause of two synchronous BCCs of the periungual tissue of two fingernails [110]. Diagnosis is often delayed, being performed after 10 years on average [111].

#### 2.5.1. Clinical Features

**Figure 11:**
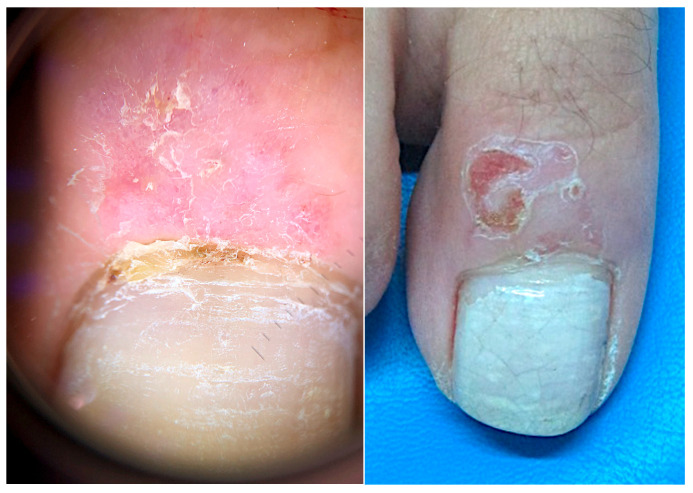
BCC of the periungual area—superficial erosions are also typical of this area (image courtesy of N.Gioia Di Chiacchio, Sao Paulo, BR).

The clinical picture may differ significantly from that observed elsewhere on the skin. The typical presentation as a nodular lesion with a pearly infiltrated border is fairly uncommon in the nail apparatus. BCC in the nail unit may exhibit a variety of clinical patterns, often with characteristics more typical of an inflammatory condition rather than a neoplastic disease: periungual BCC mimicking paronychia, chronic dermatitis, pyogenic granuloma, a bacterial or mycotic infection, amelanotic melanoma or a habit tic dystrophy have been reported (Figure 11) [112,113].

#### 2.5.2. Histopathological Features

Histopathological examination shows multiple islands and nests of basaloid cells within the superficial and mid-dermis, with a peripheral palisading and a haphazard arrangement of cells in the centers of the islands. The stroma may present fibrosis and a prominent inflammatory infiltrate mostly composed of lymphocytes and histiocytes [109].

#### 2.5.3. Treatment and Prognosis

Surgical treatment must ensure complete clearance and margin confirmation. Mohs surgery is considered the first choice in the majority of cases, with excellent functional and cosmetic outcomes [24,114]. Amputation is not indicated [106,115].

### 2.6. Malignant Onychopapilloma

Malignant onychopapilloma has been recently described as a nail unit tumor originating from the mid to distal nail matrix and extending into the nail bed [116]. Its discovery was completely accidental during surgery of a typical, although slightly painful, onychopapilloma. After the first description, four more cases followed [117,118], mostly identified after re-evaluation of former histopathological slides. All reported cases were localized on the fingernails of middle-aged patients.

#### 2.6.1. Clinical Features

The lesion presents as a classical onychopapilloma [119]: a longitudinal band of erythronychia/melanonychia with distal onycholysis and possible splitting/chipping. At present, there are no clinical signs in differential diagnosis that are useful when deciding between one form or the other. When dealing with a distal nail matrix pathology, the nail plate is focally thinned in its ventral portion, so it is easily breakable with minor trauma in its distal end. At the free edge tucked into the recess, there is a small keratinous nodule that can drop off at times or may be lost when the nail plate is cut too short. Sometimes it is scarcely visible to the naked eye and dermoscopy of the free edge is necessary [120]. As well as its benign counterpart, pigmented cases have also been described [117]. Pain seems to be the characteristic that should help with differentiating it from the benign condition, but too few cases have been reported to take it as a rule. Any unusual presentation should, however, prompt full removal. Differential diagnoses are the ones relating to onychopapilloma: fibroma/fibrokeratoma, filamentous tumor, acantholytic dyskeratotic acanthoma, onychocytic matricoma/carcinoma, nail bed acanthoma, SCC and melanoma.

#### 2.6.2. Dermoscopic Features

The lesion presents as a classical onychopapilloma [116]: a longitudinal red (erythronychia) or brown (melanonychia) band starting from the lunula and reaching the distal nail plate margin. As stated above, dermoscopy of the free edge may help in identifying hyponychial hyperkeratosis. No nail bed splinter hemorrhages have been reported so far, but their presence cannot be excluded.

#### 2.6.3. Histopathological Features

The typical alterations of onychopapilloma predominate in the nail bed, particularly in the distal part: papillomatosis of the epithelium, epithelial acanthosis, matrix metaplasia of the upper layers and hyponychial filiform distal hyperkeratosis. Conversely, in the proximal portion of the tumor, architectural disorder with cellular atypia, some atypical mitotic figures, dyskeratosis and a large proportion of round, often clear-appearing cells have been reported [116,117]. In particular, the mid-matrix shows full-thickness loss of orderly stratification and atypical cells and the distal matrix shows many clear cells and pronounced acanthotic thickening of the epithelium and papillomatosis. The distal matrix to proximal nail bed area shows clear cells, large and often vesicular nuclei, few mitotic figures and loss of orderly arrangement of the cells in most of the epithelium except the basal and parabasal layers. The proximal nail bed area shows nuclear atypia. Immunohistochemistry supports differential diagnosis, as malignant onychopapilloma exhibits an abnormal Ki67 expression and an abnormal p53 expression, which are absent in its benign counterpart. The absence of p16 expression confirms that HPV does not play a role in the etiopathogenesis [117]. Recently, nail clipping has been proposed to guide the diagnosis, being less invasive than excision. Only one case is reported, showing irregular, abnormal keratinocytes and thinning of the nail plate [121].

#### 2.6.4. Treatment and Prognosis

Due to the paucity of published cases, no data regarding long-term prognosis of malignant onychopapilloma are available to date. For this reason and due to the unknown potential of metastatization, it has been proposed that the term “atypical” is better than “malignant” [118]. Longitudinal en bloc removal of the lesion with the nail plate in place, besides assuring a satisfactory specimen for detailed histopathologic examination, showed a very good cosmetic and functional outcome and no recurrence has been observed to date [116,118]. Mohs surgery has been recently proposed as a promising option [119].

### 2.7. Malignant Onycholemmal Cyst

Malignant onycholemmal cyst is an unusual tumor of the nail bed, probably originating from a pre-existing subungual keratinous cyst. Only one report has been published to date [122]. Differential diagnosis includes viral verruca, SCC, subungual keratoacanthoma, melanoma and a benign proliferating onycholemmal cyst.

#### 2.7.1. Clinical Features

The lesion has been described as a rapidly growing, warty subungual mass, with swollen and erythematous surrounding tissues that may infiltrate the underlying bone. Pain may be reported due to the fast subungual growth pressing onto the bone [122].

#### 2.7.2. Histopathological Features

A cystic structure involving the entire nail bed, the proximal and lateral nail folds to the phalangeal bone, filled with eosinophilic, amorphous keratin and lined by slightly atypical squamous epithelium devoid of a granular layer was described. Several collections of atypical keratinocytes filling the dermis and penetrating the nail bed epithelium were also observed. Atypical cells were found to be weakly positive for cytokeratins and focally positive for epithelial membrane antigen (EMA) [122].

#### 2.7.3. Imaging

It is advisable to perform an X-ray of the affected digit before any explorative surgery to exclude possible bone invasion (osteolytic process).

#### 2.7.4. Treatment and Prognosis

The only case described to date manifested over several years as a slowly growing subungual mass, which abruptly became a warty lesion occupying more than half of the nail bed [122]. After an initial curettage with partial removal of the nail plate, the lesion recurred, growing rapidly and inducing pain. After diagnostic biopsy, the patient underwent disarticulation of the involved phalanx. More data regarding the risks of metastasis and recurrence rate are needed to investigate surgical options.

### 2.8. Onycholemmal Carcinoma

Onycholemmal carcinoma is a rare, often misdiagnosed, malignant epithelial tumor originating from the nail bed epithelium, with less than 10 cases reported in the literature. It cannot be diagnosed based on its clinical presentation because it is not specific.

#### 2.8.1. Clinical Features

**Figure 12:**
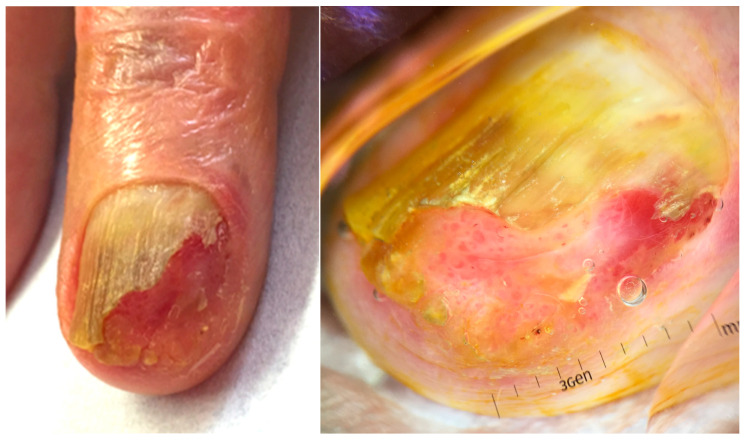
Onycholemmal carcinoma resembling an SCC. Pathology will be diriment.

Onycholemmal carcinoma presents as a slow-growing nodular mass with onycholysis and onychodystrophy to complete destruction of the overlying nail plate, appearing in both fingernails or toenails of elderly patients, more frequently in males [123,124,125,126,127,128,129]. Oozing, ulceration and bleeding are common, with erythema and swelling of the periungual tissues and variable pain (Figure 12). Clinical differential diagnosis includes SCC and amelanotic melanoma.

#### 2.8.2. Histopathological Features

Onycholemmal carcinoma presents as an infiltrative proliferation of atypical keratinocytes arranged in nests and keratinous cysts typically devoid of a granular layer [123]. This peculiar finding is defined as “onycholemmal keratinization”, which is histologically analogous to trichilemmal keratinization usually found in the midportion of the hair follicle and in trichilemmal cysts. Cystic configuration is not really a feature of SCC; however, some authors consider onycholemmal carcinoma as a subtype of SCC [124]. Differential diagnosis with a malignant onycholemmal cyst could be very subtle: they share a peculiar type of keratinization, but the latter entity usually presents with clear cells and a greater number of cystic structures [125]. Negativity for p53 and positivity for p16 at immunohistochemistry have been associated with an elevated mitotic activity and a higher proliferation index, which may suggest a more aggressive behavior [126].

#### 2.8.3. Imaging

The majority of cases reported show radiographical evidence of bone invasion [128].

#### 2.8.4. Treatment and Prognosis

Onycholemmal carcinoma exhibits the tendency to ulcerate and penetrate the phalangeal bone; however, it seems to have a low metastatic potential [124,127,128]. No standard treatment for onycholemmal carcinoma has been established, due to the paucity of cases reported. The majority of cases underwent amputation of the affected phalanx due to bone invasion, and no instances of recurrence were observed at follow-up [123,124,125,127,128,130]. Due to the indolent clinical course of the tumor, conservative surgery and radiotherapy could be considered as an alternative in cases of limited disease. Further studies on larger series of patients are required for the correct approach to this condition.

## 3. Conclusions

Malignant epithelial nail unit tumors are conditions that can significantly affect both the appearance and function of the nail but are rarely life-threatening conditions. Early diagnosis is crucial. Most of these tumors can mimic benign conditions, emphasizing the importance of clinical suspicion and histopathological evaluation when a conclusive clinical diagnosis is not possible. A comprehensive understanding of the clinical presentation, dermoscopic features and appropriate biopsy techniques is essential for dermatologists and pathologists to ensure accurate diagnosis and effective treatment.

## Figures and Tables

**Table 1:** MRI findings compared to HFUS and RCM in the approach of SCC.

MRI Findings	HFUS Findings	RCM Findings
Low-intensity signal on T1 weighted images and high-intensity signal on T2 * Well-defined deep margin in Bowen and blurred one in invasive SCC Possible bone edema/invasion Heterogeneous enhancement after contrast medium (gadolinium) injection Three-dimensional images and biomodels to better visualize the lesion’s extent and relationship with nearby structures (only in selected centers)	Heterogeneously hypoechoic mass with a solid component Possible irregular and infiltratrive margins Possible focal anecoic or hypoecoic necrosis Possible cortical bone erosion Hypervascular pattern on color Doppler	Distal matrix: small, bright, non-nucleated cells indicative of pigmented keratinocytes with a cobblestone appearance + rare small dendritic cells Proximal bed: roundish structures with small bright non-nucleated cells and central keratinization, corresponding to the epithelial nests infiltrating the upper dermis

* This is not a specific finding, since it can also be found in glomus tumors.
